# ^18^F-Fluoride PET/CT Imaging of Medication-Related Osteonecrosis of the Jaw in Conservative Treatment—A Case Report

**DOI:** 10.3389/fonc.2021.700397

**Published:** 2021-07-01

**Authors:** Christian Philipp Reinert, Christina Pfannenberg, Sergios Gatidis, Christian la Fougère, Konstantin Nikolaou, Sebastian Hoefert

**Affiliations:** ^1^ Department of Radiology, Diagnostic and Interventional Radiology, University Hospital Tübingen, Tübingen, Germany; ^2^ Department of Radiology, Nuclear Medicine, University Hospital Tübingen, Tübingen, Germany; ^3^ Cluster of Excellence iFIT (EXC 2180) “Image Guided and Functionally Instructed Tumor Therapies, ” University of Tübingen, Tübingen, Germany; ^4^ German Cancer Consortium (DKTK), Partner Site Tübingen, Tübingen, Germany; ^5^ Department of Oral and Maxillofacial Surgery, University Hospital of Tübingen, Tübingen, Germany

**Keywords:** PET/CT, ^18^F-fluoride, hybrid imaging, medication-related osteonecrosis of the jaw, bisphosphonates, antiresorptive therapy

## Abstract

Medication-related osteonecrosis of the jaw (MRONJ) is a serious side effect in antiresorptive treatment. Treatment of MRONJ is considered primarily conservative with oral mouth rinses and antibiotics but may demand surgery, depending on the complaints and general condition of the patient, the extent of the necrosis, and the overall prognosis with respect to the underlying disease. A 77 year old female patient with invasive ductal breast cancer and bone metastases was treated with intravenous bisphosphonate (BP) zoledronic acid. During therapy, she developed MRONJ in the mandible with severe pain. Clinical examination revealed confluent exposed bone of the lower left jaw and a fistula at the right molar region. The panoramic radiograph revealed a mandibular osseous involvement with diffuse radiopaque areas between radiolucent areas. For preoperative planning, ^18^F-fluoride positron emission tomography/computed tomography (PET/CT) of the jaw was performed, showing substantially increased ^18^F-fluoride uptake in regions 38 to 47 of the mandible with a focal gap in region 36 (area of clinically exposed bone). CT revealed medullary sclerosis and cortical thickening with confluent periosteal reaction and focal cortical erosion in the regions 37 to 42, whereas the regions 43 to 47 were only subtly sclerotic without cortical thickening. After systemic antibiotic therapy with sultamicillin following significant symptom and pain relief, ^18^F-fluoride PET/CT imaging was performed again after 5 months. No changes in either CT and PET were observed in regions 38 to 42, whereas the bony sclerosis was slightly increased in regions 43 to 47 with a slight reduction of ^18^F-fluoride uptake. ^18^F-fluoride PET/CT showed no significant changes assessing the extent of MRONJ prior and after systemic antibiotic therapy, providing no evidence that conservative treatment reduced the extent of the MRONJ-affected jawbone. The additional information of ^18^F-fluoride PET enables to identify the true extent of MRONJ which may be underestimated by CT imaging alone. Patients with MRONJ undergoing conservative treatment could benefit because additional imaging may be avoided as the pre-therapeutic ^18^F-fluoride PET/CT delivers all information needed for further treatment. Our findings support the recommendation of a surgical approach as long-term antibiotics cannot downsize the extent of MRONJ.

## Introduction

Medication-related osteonecrosis of the jaw (MRONJ) is a serious side effect in antiresorptive treatment and does have an enormous impact on quality of life of affected patients. Bisphosphonates (BP) and the monoclonal antibody denosumab (DNO) against the receptor activator of nuclear factor kappa-B ligand (RANKL) are inhibitors of osteoclastic bone resorption (1–3).

If administered over a long term and/or at high doses, BP are known to increase the risk of MRONJ (4). DNO is considered to increase the risk, too (3). MRONJ is defined by an area of exposed jawbone or bone that can be probed through at least one intraoral or extraoral fistula persisting for more than 8 weeks, and no history of radiation therapy or obvious metastatic disease of the jaw (5). So far, the specific etiology of MRONJ is not entirely clear and multiple hypotheses like inhibited osteoclast differentiation, increased osteocyte apoptosis, decreased bone resorption, and diminished remodeling are being discussed (6). The effects of antiresorptive agents on bone metabolism are considered site-specific, however, it is still not completely clear why MRONJ develops in the jaw.

In recent years, a substantial increase in the prevalence of MRONJ has been observed as affiliated drugs are currently being used in various clinical applications (7). As defined by the American Association of Oral and Maxillofacial Surgeons (AAOMS), MRONJ is currently classified into four stages (8). Patients with stage 0 have no evidence of necrotic bone but present with nonspecific clinical findings, radiographic changes, and symptoms, whereas patients who are classified as stage 1 present with exposed and necrotic bone without signs of infection. Patients with stage 2 suffer from clinical symptoms such as pain and erythema and signs of infection. Patients with stage 3 present with necrotic bone beyond the region of alveolar bone and complications such as pathologic fractures or extra-oral fistula (8).

Clinical examination of the jaw in combination with panoramic radiography (orthopantomographs), and more recently, cone-beam computed tomography (CBCT) for comprehensive three-dimensional morphological characterization of the jawbone, are considered as the standard preoperative work-up of patients with MRONJ to provide initial information about the bone structure, course, extent, and progression of the disease (3, 9). In advanced stages or before surgical intervention, the use of multi-detector CT (MDCT), magnetic resonance imaging (MRI), or bone scintigraphy is useful to determine the extent of the disease, guide therapeutic options, and monitor the treatment response (10).

For treatment success, it is essential to accurately define the bony margins for the planned surgery preoperatively. Although CT and MRI have been reported to have a high diagnostic accuracy for detection of MRONJ that exceeds that of panoramic radiographs, both techniques are limited in exactly assessing the extent of disease (11). ^18^F-fluoride PET/CT allows technicians to detect subtle foci of increased bone remodeling depicting larger areas of MRONJ than conventional CT imaging or preoperative clinical examination (12).

We report a case of MRONJ caused by long-term application of BP and DNO, which was examined twice with ^18^F-fluoride PET/CT, prior and after systemic antibiotic treatment for 45 days. The aim of this case report is to demonstrate the feasibility of ^18^F-fluoride PET/CT to estimate the changes of MRONJ-affected jawbone prior and after long-term conservative treatment.

## Methods

### ^18^F-Fluoride PET/CT Imaging

Both ^18^F-fluoride PET/CT examinations were performed on a state-of-the-art clinical scanner (Biograph mCT, Siemens Healthineers). PET/CT imaging started 60 min after intravenous application of 320 MBq of ^18^F-fluoride. The patient was positioned in a vacuum mattress to reduce beam-hardening artifacts and motion artifacts. CT examination was performed without a CT contrast agent, using a bone image reconstruction kernel and a slice thickness of 0.6 mm for image reconstruction. PET was acquired from the skull base to the clavicle over one bed position and reconstructed using a 3D ordered subset expectation maximization algorithm (two iterations, 21 subsets, Gaussian filter 2.0 mm, matrix size 400 × 400, and slice thickness 2.0 mm). The PET acquisition time was 2 min.

### Image Analysis

Image analysis of CT and PET images was performed both quantitatively and qualitatively.

In PET, the amount of ^18^F-fluoride uptake was evaluated for each region of the jawbone. Regions showing substantially increased or decreased ^18^F-fluoride uptake above the background radiotracer activity of healthy bone structures were documented. In CT, the bone structure was evaluated in terms of periosteal thickening, focal erosions, or medullary sclerosis.

For quantitative analysis, CT-Hounsfield Units (HU) of the jawbone were measured using two-dimensional regions of interest (ROIs) in each region of the mandible, carefully excluding the cortical bone and the teeth. For each CT ROI, mean attenuation numbers (Hounsfield Units [HU]) with standard deviation (SD) were recorded. The mean HU of the MRONJ-affected jaw was calculated by the mean of all affected regions. Correspondingly, the mean HU of the healthy jawbone was calculated by the mean of all regions not affected by MRONJ. To calculate a semi-quantitative index for both examinations, mean HUs of the MRONJ-affected jawbone were divided by the mean HUs of the healthy jawbone.

Correspondingly, PET-standard uptake values (SUV) of the jawbone were measured using 50% isocontour volumes of interest (VOIs) in each region of the mandible. For each PET VOI, SUV_mean_ with SD was recorded. The mean SUV of the MRONJ-affected jawbone and healthy jawbone as well as the calculated semi-quantitative index for both examinations were documented.

## Case Report

A 77-year-old female patient with invasive ductal breast cancer presented with MRONJ of the mandible in April 2014 in our department. After initial diagnosis in November 2000, she developed bone metastases and had a pathological right femoral neck fracture. In February 2001, she received adriamycin and taxotere in a neoadjuvant setting, followed by breast conserving surgery and lymphonodectomy and finally cyclophosphamide, methotrexate, and fluorouracil (CMF) chemotherapy combined with exemestane. Further, she developed recurrent sintering fractures of thoracic and lumbar vertebral bodies caused by severe osteoporosis followed by multiple stabilizing surgeries.

### Clinical Findings

As oral antiresorptive therapy, she received bondronate from March 2001 to August 2003, followed by intravenous application until November 2013. This was followed by subcutaneous application of 120 mg DNO monthly from December 2013 to July 2014 and in September 2014 as a single application. In April 2014, she showed exposed oral jawbone in region 38 with a size of 4 × 3 mm and region 37 with a size of 2 × 1 mm at the lingual rim of the mandible. She complained about pain and showed signs of moderate infection (stage 2 after Ruggiero). After 3 days of antibiotic therapy, she reported pain relief. Antibiotics were then continued for 3 weeks. After 3 weeks, no signs of local infection were evident (stage 1). Additionally, local mouth rinses with chlorhexidine 0.1% once a day and green tea were recommended.

Because of necessary oncological treatment, DNO was continued in November 2014 with exemastane medication, again. Until 2018, she continued with local mouth rinses. Due to acute pain and signs of boosted infection, sultamicillin short-term therapy was conducted. For a total of nine instances, a short-term antibiotic therapy was necessary, however, the patient still preferred conservative treatment over surgical therapy.

**Figure 1:**
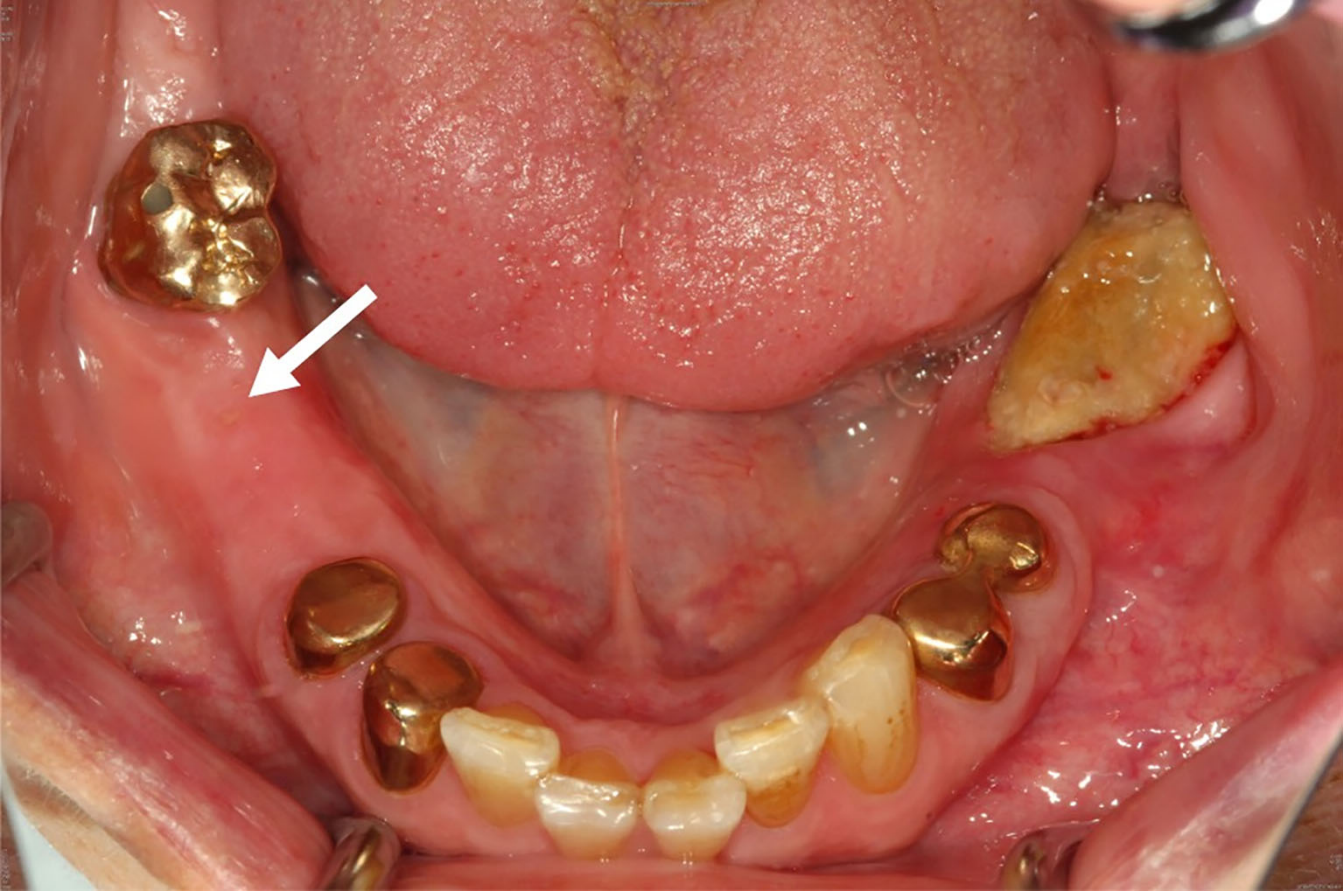
Clinical presentation of the MRONJ showing bone exposure in regions 36 to 38 and a fistula and palpable bone in region 46 (white arrow).

**Figure 2:**
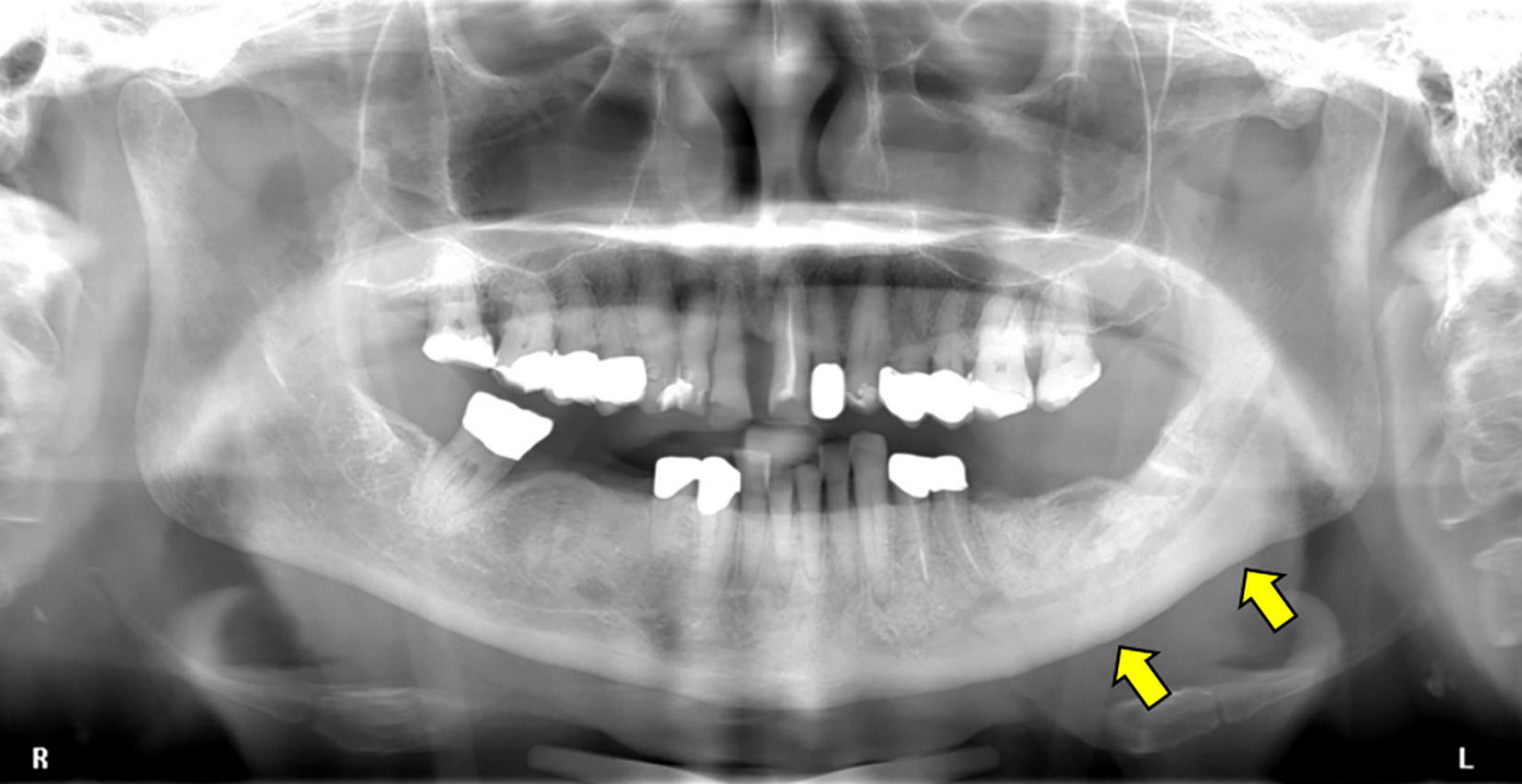
Panoramic radiograph showing sclerosis of the left mandible (yellow arrows) surrounding the inferior alveolar canal. No evidence of sclerosis in the right mandible.

**Figure 3:**
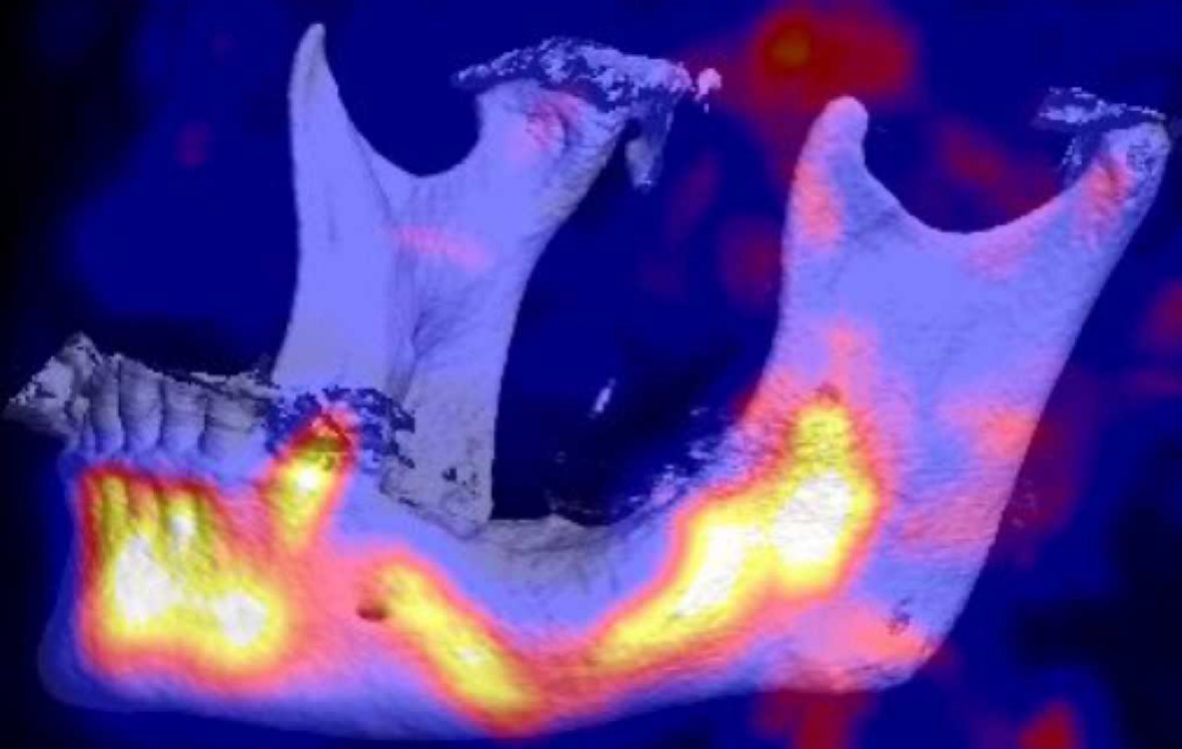
Baseline ^18^F-fluoride PET/CT VRT (Volume Rendering Technique) 3D reconstruction of the jawbone showing substantially increased ^18^F-fluoride uptake in the regions 38 to 47 of the mandible with a focal gap in region 36.

In April 2018, clinically confluent exposed bone of the lower left jaw and a fistula at the right molar region was visible (**Figure 1**). The panoramic radiograph revealed a mandibular involvement with diffuse radiopaque areas between radiolucent areas (**Figure 2**). For preoperative planning, we performed a ^18^F-fluorine-PET/CT of the jaw, showing stage 3 after Ruggiero (**Figure 3**).

**Figure 4:**
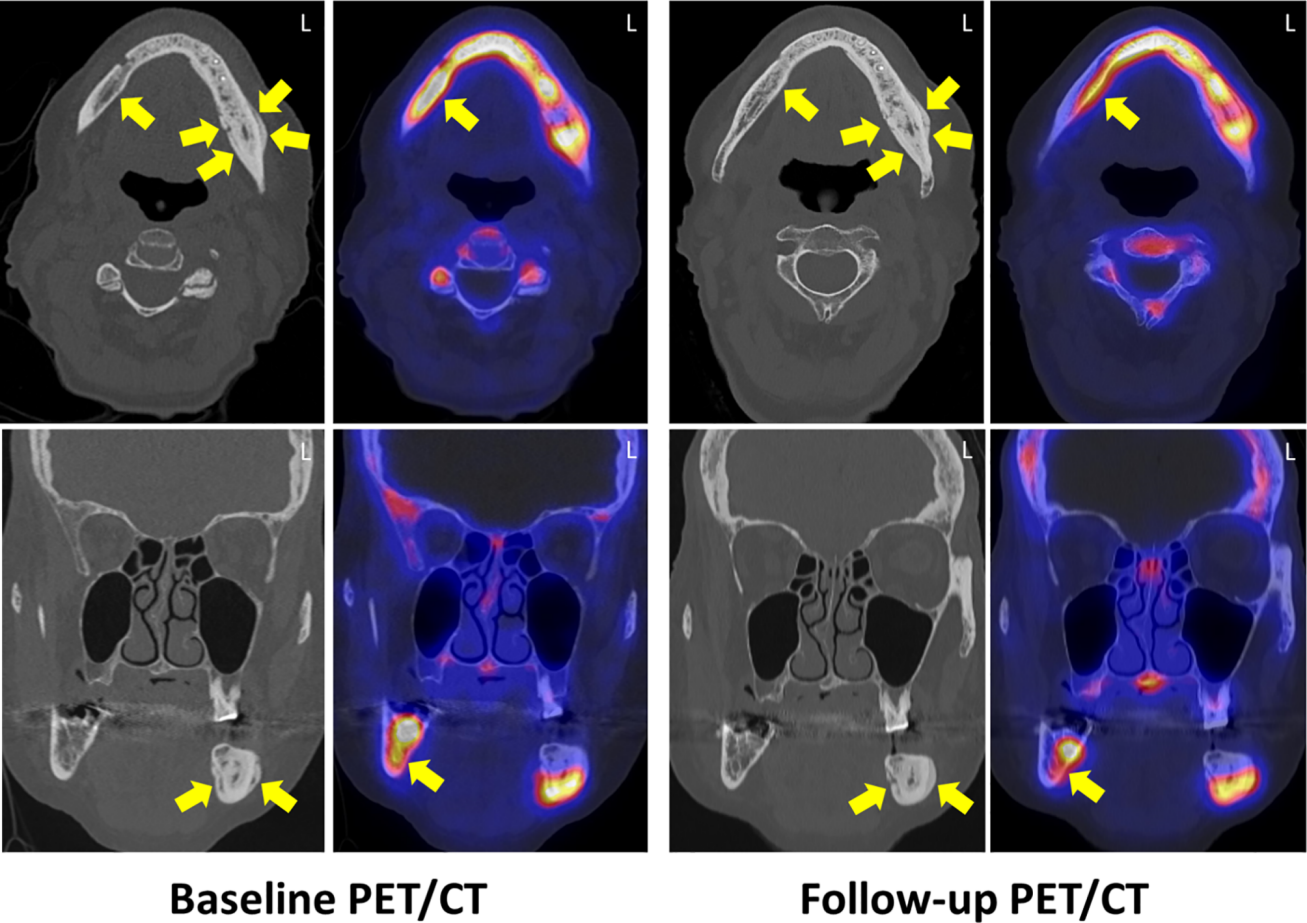
Baseline and follow-up ^18^F-fluoride PET/CT examination 45 days after systemic antibiotic therapy. Axial (upper row) and coronal (lower row) CT reveals diffuse medullary sclerosis and a cortical thickening with confluent periosteal reaction (regions 37 to 42) and a focal cortical erosion (yellow arrows). The inferior alveolar canal on the left side is relatively narrowed compared to the right side. PET reveals substantially increased ^18^F-fluoride uptake in the regions 38 to 47 with a focal gap in region 36, whereas in CT, the regions 43 to 47 were only subtly sclerotic without cortical thickening. In follow-up, no changes in both CT and PET were observed in regions 38 to 42, whereas the bony sclerosis was slightly increased in regions 43 to 47 with a slight reduction of ^18^F-fluoride uptake. L, left site.

The patient was admitted to the hospital and received 3 g of ampicillin/sulbactam three times a day combined with metronidazole 500 mg three times a day. After 3 days, pain was relieved and the patient further denied surgery. After 4 days, antibiotics were applied orally (sultamicillin) and the patient was discharged. Antibiotics were continued for 45 days in total. A follow-up ^18^F-fluorine PET/CT was performed in September 2018 at clinical stage 1 (**Figure 4**). Conservative treatment with local mouth rinses was continued until April 2019 (1 year), when an extraoral fistula appeared at the left mandible. Observation followed until June 2020 and follow-up was paused because of COVID-19 infection risk. So far, the clinical situation was stable with low infection signs and moderate purulent discharge at the extraoral fistula. DNO was paused in November 2019, exemestane continued.

### Qualitative Image Analysis

The first ^18^F-fluoride PET/CT in May 2018 showed a substantially increased ^18^F-fluoride uptake in the regions 38 to 47 of the mandible with a focal gap of ^18^F-fluoride uptake in region 36 (area of clinically exposed bone) (**Figure 4**). CT revealed diffuse medullary sclerosis and cortical thickening with confluent periosteal reaction and focal cortical erosion in the regions 37 to 42. The regions 43 to 47 were only slightly sclerotic without cortical thickening, periosteal ossification, or cortical erosion. Sequestration was not found.

Follow-up ^18^F-fluoride PET/CT imaging was performed in November 2018. Whereas no changes in both sclerosis and ^18^F-fluoride uptake were observed in the regions 38 to 42; the regions 43 to 47 showed a slight reduction of ^18^F-fluoride uptake and a slightly increasing diffuse medullary sclerosis and periosteal thickening (**Figure 4**).

### Quantitative Image Analysis

At baseline ^18^F-fluoride PET/CT in May 2018, we measured 906 ± 221 HU in the MRONJ-affected regions 38 to 47, whereas 949 ± 173 HU was measured at follow-up. The mean HU of the healthy jawbone was 232 ± 76 HU at baseline ^18^F-fluoride PET/CT and 220 ± 63 HU at follow-up ^18^F-fluoride PET/CT, respectively. Using the sum of all MRONJ-affected regions and healthy bone regions, we calculated a semi-quantitative HU index of 3.9 at the baseline examination and 4.3 at the follow-up examination.

The SUV of the MRONJ-affected jawbone was 12.4 ± 1.5 at baseline ^18^F-fluoride PET/CT and 9.9 ± 1.4 at follow-up. The mean SUV of the healthy jawbone was 2.0 ± 0.3 at baseline examination and 2.0 ± 0.4 at follow-up examination. In PET, the calculated semi-quantitative index was 6.20 at the baseline ^18^F-fluoride PET/CT and 4.95 at the follow-up ^18^F-fluoride PET/CT.

## Discussion

Treatment of MRONJ is difficult and can be performed conservatively or surgically (1, 13). Both concepts have advantages and disadvantages regarding quality of life and sustainable therapy success (1–3, 13). In this case report, we describe clinical and hybrid imaging findings in a patient with breast cancer and MRONJ undergoing ^18^F-fluoride PET/CT prior and after long-term antibiotic treatment. Before initiating therapy, PET/CT revealed diffuse medullary sclerosis and periosteal reaction in the affected regions of the jaw and a substantially increased ^18^F-fluoride uptake extending beyond the conspicuous regions in CT. After systemic treatment and symptom relief, follow-up ^18^F-fluoride PET/CT delivered stable results with moderately increasing medullary sclerosis and a slight reduction of ^18^F-fluoride uptake in a region that was initially not conspicuous in CT.

Our observation that MRONJ is characterized by sclerotic changes, periosteal reaction, and cortical destruction is in concordance with the literature (14–17). BP suppress bone remodeling by targeting osteoclasts and disrupting their function (18). The alteration in material properties of the bone allows microdamage accumulation, that affect the biomechanical integrity (19). In adults, the predominant site of remodeling is the periosteal bone surface, where formation modeling serves to biomechanically offset the loss of bone adjacent to marrow that occurs with aging and estrogen deficiency (19). Therefore, BP increases sclerosis or periosteal thickening beyond the clinically overt MRONJ. Medullary sclerosis, characterized by disorganized microtrabeculae and poor corticomedullary differentiation in the affected site, is predominant in advanced stages of MRONJ and has been described as an imaging finding (14, 20). Although radiographs are typically employed as the first line in routine radiologic evaluation providing primary information after clinical diagnosis for primary diagnosis of MRONJ, the sensitivity with respect to small lesions or the extent of lesions is limited (21, 22). ^99m^Tc bone scintigraphy and ^18^F-fluorodeoxyglucose (FDG)-PET/CT have been proven to detect changes of bone metabolism caused by early onset of MRONJ (23, 24). However, a major limitation of bone scintigraphy is its limited anatomical information, whereas both scintigraphy and ^18^F-FDG-PET/CT have less sensitivity compared to ^18^F-fluoride bone scan (25, 26).

In the presented case, MRONJ was accompanied with substantially increased uptake of the radiotracer ^18^F-fluoride. This observation has been also described by Guggenberger et al. (12). As ^18^F-fluoride is incorporated in hydroxyapatite by osteoblasts during bone neoformation, it allows for detection of subtle foci of increased bone remodeling, which are considered precursors to clinically overt MRONJ (27, 28). This may also be an explanation for our observation that the extent of ^18^F-fluoride uptake overlapped the conspicuous area in CT, indicating that the bone metabolism is altered beyond the morphologically visible MRONJ and can be visualized by ^18^F-fluoride uptake. Necrotic bone can be identified by a focal lack of ^18^F-fluoride accumulation. This is in concordance with the findings of Guggenberger et al. and may be of relevance for planning potential surgical resection (12).

In a focal necrotic region of the left mandible with adjacent periosteal ossification, ^18^F-fluoride uptake was correspondingly decreased. An explanation is that areas of necrosis are accompanied with locally decreased bone metabolism due to the absence of blood supply. This feature has been also observed in bone scintigraphy scans showing significant decreases of radionuclide activity (^99m^Tc-MDP uptake) in necrotic areas (15). However, due to its limited specificity and the lack of anatomical data, scintigraphy cannot clearly differentiate if the uptake occurs within the MRONJ lesion or within the surrounding reactive bone (16). Although this limitation can be partly overcome by the use of single photon emission/computed tomography (SPECT), ^18^F-fluoride PET/CT offers increased sensitivity and specificity with shortened examination time, which is related to the coincidence-detection method to acquire photons without need of a collimator and the favorable kinetic characteristics of ^18^F-fluoride resulting in higher bone-soft tissue contrast (29).

In CT, necrotic foci are associated with a loss in bone mineralization and fragmentation of bone leading to decreased attenuation (30). The combination of CT and PET in one examination considerably facilitates the anatomical localization of metabolic changes. **Table 1** summarizes the imaging characteristics of MRONJ in ^18^F-fluoride PET/CT and panoramic radiograph compared to MRI, ^99 m^Tc bone scintigraphy/SPECT, and ^18^F-FDG PET/CT.

**Table 1:** Imaging characteristics of MRONJ in ^18^F-fluoride PET/CT and panoramic radiograph compared to MRI, ^99m^Tc bone scintigraphy/SPECT, and ^18^F-FDG PET/CT.

Imaging modality	Imaging characteristics of MRONJ
Panoramic radiograph	diffuse sclerosis showing radiopaque areas between radiolucent areas
^18^F-fluoride PET/CT	diffuse medullary sclerosis and cortical thickening with confluent periosteal reaction focal necrotic regions and cortical erosions with adjacent periosteal ossification ^18^F-fluoride uptake increased due to bone remodeling (overlapping the conspicuous sclerotic area in CT) ^18^F-fluoride uptake decreased in necrotic areas of the bone
MRI (10, 14)	variable signal intensity depending on the disease stage hypointense on T1-weighted images and iso- or hyperintense on T2-weighted images in the case of sequestrum, low signal intensity in both T1- and T2-weighted images increased gadolinium enhancement in the necrotic area and adjacent bone inflammation-induced gadolinium enhancement of the adjacent soft tissue
^99 m^Tc bone scintigraphy / SPECT (15, 16, 29)	^99m^Tc-MDP uptake increased with a focal decrease in necrotic areas of the bone lower bone-soft tissue contrast compared to ^18^F-fluoride PET**/**CT limited sensitivity/specificity
^18^F-FDG PET (25, 26)	increased ^18^F-FDG uptake (limited sensitivity) inflammatory bone and soft tissue disorders are also characterized by an increased ^18^F-FDG uptake (limited specificity)

In this clinical case, ^18^F-fluoride PET/CT delivered stable results for assessing the extent of MRONJ both prior and after systemic antibiotic therapy of 45 days, whereas a significant symptom relief was reported by the patient.

Clinical findings of MRONJ such as localized pain, soft tissue swelling, loosened teeth, and exposed bone, as well as imaging findings are, however, non-specific as they are also found in other conditions such as osteomyelitis, osteoradionecrosis, or cancer metastases (17).

The stage of MRONJ at presentation is prognostic for the success of treatment with a very low healing probability at advanced stages, therefore, early detection before the development of stage 2 has gained importance (31). Conservative treatment is recommended in stages 1 and 2, although cumulative curative rates are considered lower (0–17.2%) compared to extended surgery (4.7–97%) (1–3, 13). However, with conservative treatment it is possible to stabilize the disease, reduce symptoms, and it may be preferable in patients whose reduced general condition does not allow for surgery or who reject surgery (13).

Until now, no general consensus exists whether long-term antibiotics can stabilize and downsize MRONJ permanently. In the presented case, long-term antibiotic therapy slightly reduced the intensity of radioactive tracer uptake but not the extent of MRONJ-affected bone. *Vice versa*, it seems that the stage and therefore the intensity of inflammation do not correlate with the size of MRONJ.

According to our findings, patients with MRONJ undergoing long-term antibiotic therapy could benefit because additional imaging procedures before a later surgical approach may be avoided as the pre-therapeutic ^18^F-fluoride PET/CT delivers all necessary information needed for further treatment. This case highlights the reliability of ^18^F-fluoride PET/CT as an imaging modality to stage the extent of MRONJ independently from clinical findings and symptom relief in the course of long-term antibiotic treatment. Our findings also support the recommendation of an early surgical approach of the German guideline “Antiresorptive Drug-related Osteonecrosis of the Jaw” (ARONJ) as long-term antibiotics cannot downsize the extent of MRONJ.

In conclusion, the presented case shows that ^18^F-fluoride PET/CT is useful for estimation of the extent of MRONJ independently from established clinical stages of disease. It is noteworthy that conservative treatment may affect the severity of disease and improve symptoms, but may not reduce the size of MRONJ. This is concordant with the follow-up imaging results of this patient developing a flare-up of infection after one year. Obviously, further studies are necessary to validate this first observation.

## Data Availability Statement

The original contributions presented in the study are included in the article/supplementary materials. Further inquiries can be directed to the corresponding author.

## Ethics Statement

The studies involving human participants were reviewed and approved by the Ethics Committee at the Faculty of Medicine, University of Tübingen, Gartenstraße 47, 72074 Tübingen. The patients/participants provided their written informed consent to participate in this study. Written informed consent was obtained from the individual(s) for the publication of any potentially identifiable images or data included in this article.

## Author Contributions

CR, SH, SG, and CP contributed to conception and design of the study. CR and SH organized the database. CR wrote the first draft of the manuscript and designed the figures. SH wrote sections of the manuscript. CF and KN supervised the project. All authors contributed to the article and approved the submitted version.

## Funding

This study is supported by the Junior Clinician Scientist Program of the Medical Faculty of Tübingen.

## Conflict of Interest

The authors declare that the research was conducted in the absence of any commercial or financial relationships that could be construed as a potential conflict of interest.
